# Validation of a digital, partly automated three-dimensional cast analysis for evaluation of orthodontic treatment assessment

**DOI:** 10.1186/s13005-025-00515-8

**Published:** 2025-05-08

**Authors:** Franziska A. Lang, Norbert A. Lang, Julia Vorloeper, Christian Niederau, Rogerio B. Craveiro, Isabel Knaup, Michael Wolf

**Affiliations:** Department of Orthodontics, University Hospital RWTH Aachen, Pauwelsstraße 30, 52074 Aachen, Germany

**Keywords:** Digital model analysis, Comparison digital analogue, Partly automated, Analysis software

## Abstract

**Background:**

Plaster models have been considered the gold standard in traditional orthodontic model analysis. Modern imaging techniques and ever-advancing technologies have expanded the scope of digital diagnostic tools. These innovations allow the use of devices specifically designed for the diagnosis of 3D structures. The aim of this method comparison study was to determine the accuracy and efficiency of digital measurements compared to conventional manual measurements on plaster models.

**Materials and methods:**

The present cohort constitutes the evaluation of pretherapeutic situation models of 247 orthodontically treated patients (129 females and 118 males, average age 16.76 +- 9.49 years) with mixed or permanent dentition who were treated at the University Hospital RWTH Aachen between January 2018 and December 2020. Plaster models were digitised using a model scanner, and an experienced examiner performed various measurements on blinded plaster models using a calliper and on digital models using the specially developed ‘Tooth width analysis Aachen’ patch in the OnyxCeph3TM-3D software. The intra-rater and inter-rater reliability were determined by a second, blinded assessor. Measurements included tooth width, crown height, arch width, arch length and arch circumference, as well as overjet and overbite. Differences between analogue and digital methods were calculated.

**Results:**

Differences of up to 0.3 mm were observed between manual and partially automated digital measurements for sagittal, transversal and vertical parameters. Teeth with close proximal contact to adjacent teeth and teeth in jaws with a negative space analysis result showed an increased difference between manual and partially automated digital measurements, although this was not clinically relevant. The time required to perform digital measurements was statistically significantly reduced.

**Conclusions:**

Partially automated digital impression analysis offers an accurate, highly efficient and time-saving alternative to traditional manual impression analysis.

**Supplementary Information:**

The online version contains supplementary material available at 10.1186/s13005-025-00515-8.

## Introduction

Three-dimensional model analyses, based on plaster casts of the upper and lower dentition of a patient, constitute an essential component of orthodontic diagnostics [1]. Conventionally, model analysis is performed on plaster models, which succeeded for many years in orthodontic practice [2]. Nevertheless, due to the inherent limitations of conventional plaster dental casts, including increased storage space requirements and the potential for damage due to handling, digital scanners for obtaining virtual models and several software programs for digital model analyses have been developed and disseminated in orthodontics. There are a number of methods for obtaining digital models, including scanning of the patient’s dentition using an intraoral scanner at the office, scanning of an alginate or silicone impression, scanning of a plaster model with a laboratory scanner, and scanning using Cone Beam Computed Tomography (CBCTs) following 3D imaging reconstruction [3–5]. The advent of digital technology has made it possible for both soft and hard tissues to be visually represented electronically in a modern orthodontic practice. As a consequence, digital models are emerging as the new standard for orthodontic medical documentation, potentially superseding plaster models [3, 4]. However, its validity compared to traditional model analysis using callipers remains to be assessed, especially as a variety of hardware and software solutions are available to the user. The current research situation is supported by several comprehensive systematic reviews that consistently confirm the reliability of digital workflows in clinical applications. This broad evidence base, supplemented by current validation studies, emphasises the advanced state of development of digital workflow solutions in a clinical context [3, 4, 6]. In recent times, technological advances have enabled the creation of digital three-dimensional dental models, which have gained considerable popularity [7]. This may be attributed to the numerous advantages offered by digital analysis, as outlined below. Whether it is a tooth width measurement or a Bolton analysis, the literature shows that digital model evaluation can save a significant amount of time - seconds or even minutes [7–10]. It is a common occurrence for orthodontists to encounter difficulties when attempting to store their patients’ records due to the limited space available. This is particularly problematic for those who have been in practice for several years and are required to adhere to the legal retention periods for patient records. Therefore, is the elimination of the storage problem of plaster models another advantage, which require physical storage space. In contrast, digital models can be stored efficiently on hard drives or portable storage devices. This facilitates data access, which in turn opens up new opportunities for interdisciplinary communication and collaboration [6, 11–13]. Digital methods are becoming increasingly pertinent for orthodontic cast analysis. However, many digital methods are still time-consuming due to the involvement of numerous manual steps. Consequently, the development of partially automated and self-measuring tools has become of great interest.

Therefore, as part of a retrospective cross-sectional study of orthodontically treated patient cases to validate the specially developed ‘Tooth width analysis Aachen’ patch in the OnyxCeph3TM-3D software, we wanted to compare traditional manual model analysis of plaster models and semi-automated digital model analysis to develop another gold standard for digital orthodontic model analysis to be used in everyday orthodontic practice. The existing plaster models were digitized for this purpose, while intraoral scans were not routinely available. This digital model analysis should efficiently generate precise, objective and reproducible data that provides important information on the orthodontic treatment situation; for pre-therapeutic orthodontic treatment planning, for re-evaluation as part of an interim diagnosis or post-therapeutically for comparison with the pre-therapeutic initial situation and assessment of the orthodontic treatment quality.

The primary objective of this retrospective study was to evaluate the degree of agreement and accuracy between digital and analogue model analysis. As secondary outcomes, risk factors that increase measurement deviation and the required measurement time were evaluated. To this end, a sample of 247 initial models, all in the mixed dentition phase, were analysed in both analogue and digital models.

## Material and method

### Study design

The present study employs a method-comparison approach to assess the degree of agreement between digital and analogue model analysis, as well as the required time of each method. Ethical approval was obtained from the ethics committee of Medical Faculty of RWTH Aachen with the ethical registration number EK 232/20.

### Data acquisition

The power calculation was performed using G Power 3.1 [14]. For the power analysis we used the smallest tooth, the premolar 15 and the smallest measurement method, the tooth width analysis, as the SD has the greatest effect/disadvantage here. A total of 247 pseudonymised cases were included from patients being treated during the period from January 2018 to December 2020. The case records were obtained from the archives of the University Hospital Aachen, RWTH, Germany and consist of consist of pre- treatment plaster models with inclusion criteria described in the following: (i) Good condition of dental casts (not fractured or degraded), (ii) mixed or permanent dentition (without further differentiation with regard to e.g. tooth agenesis or tooth eruption) and (iii) Irrespective of crowding, malocclusion, and previous orthodontic treatment. Plaster models of insufficient quality or damaged models with incomplete teeth that could not be evaluated were not digitised and therefore not included in the digital database.

The plaster casts were also scanned with orthoX^®^scan (Dentaurum GmbH & Co. KG, Ispringen, Germany) in accordance with the manufacturer’s instructions. Three-dimensional virtual models were subjected to visual inspection for accuracy and, where necessary, scanning was conducted until high-quality models were acquired. Subsequently, the virtual models were imported into the software *OnyxCeph*^*3TM*^ (Image Instruments GmbH, Chemnitz, Germany), which was used by one experienced investigator, familiarised with the software, for digital model analyses. Based on the studies of Coenen et al. [15, 16], the ‘Tooth Width Analysis Aachen’ patch for orthodontic model analysis was developed in collaboration with *OnyxCeph*^*3TM*^-3D for the further development and continuation of the digitalisation of orthodontic diagnostics, which is not yet freely accessible. The same plaster casts were subjected to manual analysis with a calliper (Dentaurum GmbH & Co. KG, reference number 042-751-00). A patient case consists of one upper and one lower jaw. 247 pseudonymised cases result in a total of 494 individual jaws (247 upper and 247 lower jaw), which were measured both digitally and by hand as part of the orthodontic model analysis. According to the analogue evaluation method, only the permanent teeth were measured and evaluated, but not the deciduous teeth.

### Dental cast analysis

In accordance with the definitions listed in the Table 1, the measurements and the corresponding computations represented are commonly used parameters and were used both for an analogue and digital manner. In the context of arch dimensions, the terms “anterior arch width,” “arch length,” and “arch perimeter” are used. The measurements were taken to the nearest 0.1 mm, except for overjet, which was recorded to the nearest 0.5 mm. As far as the digital measurement is concerned, the software automatically provides suggestions for reference points for all required measurements, except for the overbite and the lower posterior arch width. Nevertheless, it is necessary to review the reference points and adjust them if required (partly automated). The time required for the measurement of 50 digital (excluded the time for digitizing the plaster model) and 50 analogue cast analyses is documented.

**Table 1:** Detailed explanation of the measurements carried out with the necessary reference points for standardising the digital and analogue measurement. The measurements were only carried out on permanent teeth

Measurements	
Tooth sizes (16–26, 36–46)	Maximal mesiodistal width of the respective tooth (perpendicular to tooth axis and parallel to occlusal plane)
Crown height (12–22, 32–42)	Respective distance between the most cervical to the most incisal point (parallel to the tooth axis)
Arch perimeter (= Available space) of the maxilla (15–25)	The two lateral segments, measured from the mesial approximal contact of the first molar to the distal point of the lateral incisor on each side, should be added to the two frontal segments. These segments are defined between the distal point of the lateral incisor and the mesial point of the central incisor.
Arch perimeter (= Available space) of the mandible (35–45)	The two lateral segments, measured from the mesial approximal contact of the first molar to the distal point of the lateral incisor on each side, should be added to the two frontal segments. These segments are defined between the distal point of the lateral incisor and the mesial point of the central incisor.
Anterior and posterior arch widths maxilla	Distance between the cusps of canines and distance between the mesiobuccal cusp tips of first molars
Anterior and posterior arch widths mandible	Distance between the cusps of canines and distance between the the most gingival extension of buccal grooves of the first molar
Arch length	Distance from contact point of central incisors perpendicular to a tangent applied to the distal surfaces of first molars
Overjet	the maximum horizontal overlap of incisors
Overbite	the maximum vertical overlap of incisors
Midline deviation maxilla	Midline deviation of the upper jaw in relation to the raphe median plane
Midline deviation mandibula	Midline deviation of the lower jaw in relation to the raphe median plane
Midline deviation of the upper to the lower jaw	Midline deviation of the upper jaw in relation to the midline of the lower jaw
Duration	Time required for the aforementioned measurements to be completed (time needed for computations not included)
Computations*	
Bolton 3–3	Ratio of the sum of mesiodistal widths of teeth 33–43 and 13–33 (not applicable to early mixed dentitions)
Bolton 5–5	Ratio of the sum of mesiodistal widths of teeth 35–45 and 15–25 (not applicable to early mixed dentitions)
Upper crowding/spacing (Total difference maxilla)	Difference between the available space of the dental upper arch (arch perimeter of the maxilla) and the total sum of the mesiodistal widths of 15–25
Lower crowding/spacing (Total difference mandible)	Difference between the available space of the dental lower arch (Arch perimeter of the mandible) and the total sum of the mesiodistal widths of 35–45

*Computed from the direct measurement on the casts

### Reliability

To assess the intra- and inter-rater reliability of digital and analogue methods, 50 randomly selected pretreatment casts were subjected to repeated digital measurements, while another 50 plaster models were remeasured analogously by the same examiner after an interval of two months. Also, a second blinded examiner was briefed with the definitions of required measurements and performed 50 digital cast analyses. The results of the repeated measurements were compared between different methods and examiners. Intra- and interclass correlation coefficient was calculated with the software Python (Version 3.9.12 and pingouin library).

### Statistical analysis

**Fig. 1:**
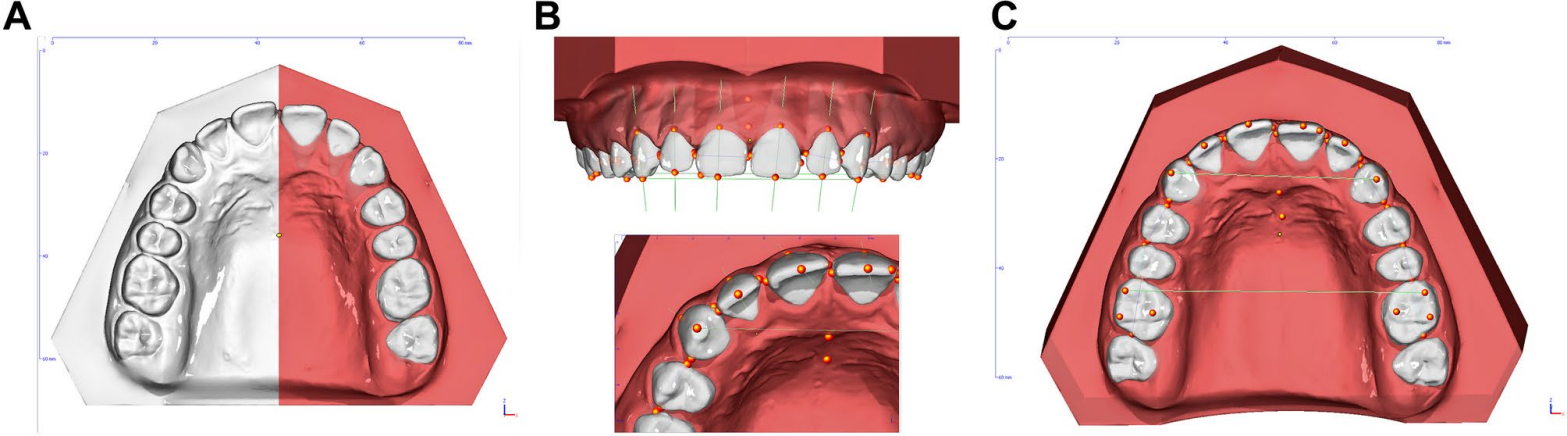
Methodological diagram of the three-dimensional digital model measurement method. **A**: Occlusal representation of the segmentation of a maxillary model to identify the areas of a closed scan surface belonging to a dental crown. **B**: Reference points are assigned to the three-dimensional dental crowns, which form an individual coordinate system and are used in the following diagnostic modules. The reference points are checked in all 3 planes. **C**: The digital model analysis is then carried out with determination of tooth widths, crown height, overjet/overbite, midline shifts, arch dimensions (anterior and posterior arch width, arch length, arch circumference)

**Fig. 2:**
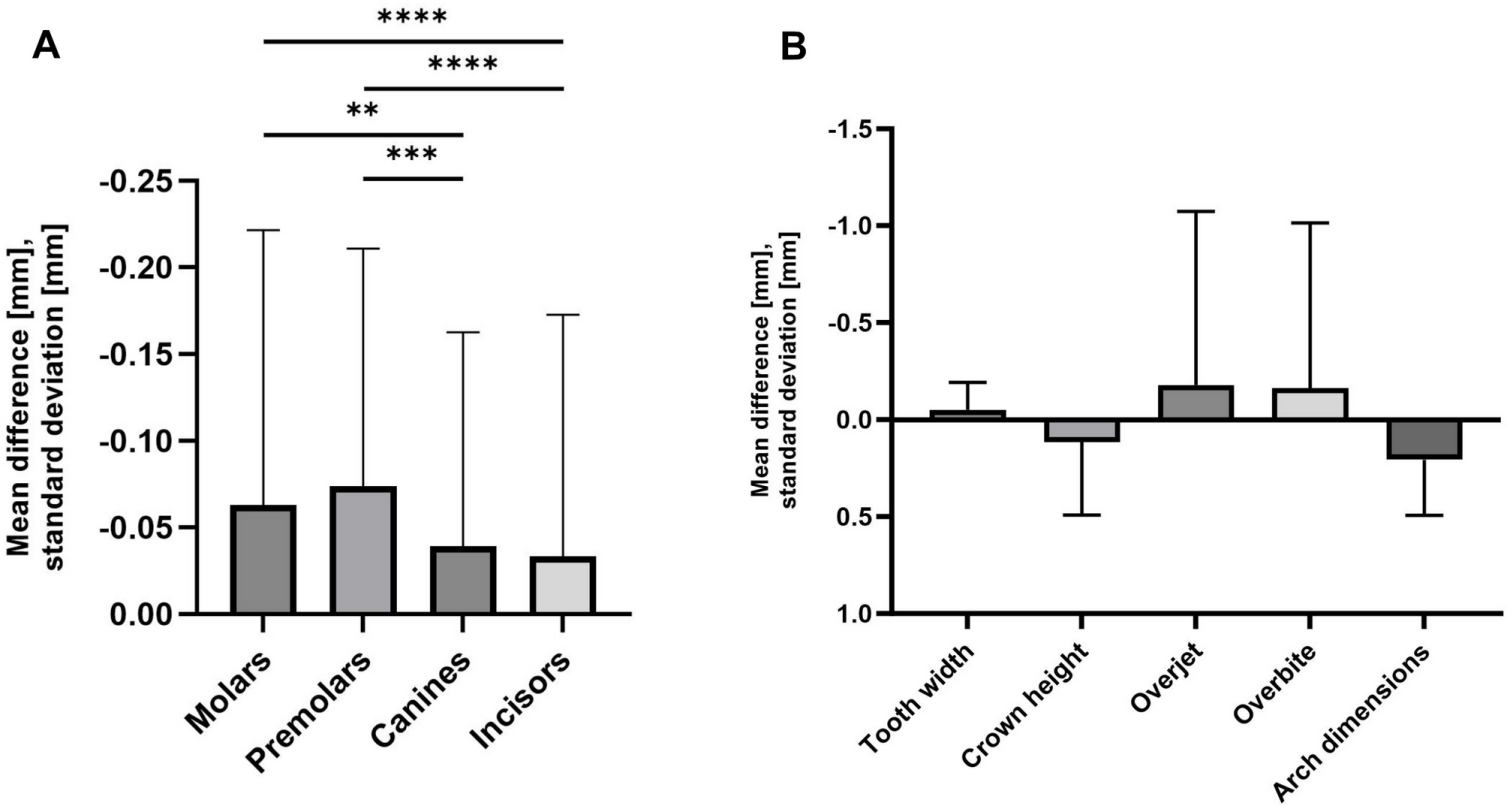
The measurement differences between the analogue and digital methods were examined in detail regarding the different tooth groups and different measurement criteria. **A**: Differences between digital and analogue measurements [mm] for the different groups of teeth. The premolars show the greatest deviation and the canines the smallest; molars IQR: 0.10, median: -0.10; premolar IQR: 0.00, median:-0.10; canines IQR: 0.00, median: -0.10; incisors IQR: -0.10, median: 0.00 **B**: A comparison of the observed absolute difference between the digital and analogue methods for tooth width (IQR: -0.10, median: 0.00), crown height (IQR: 0.40, median: 0.20), overjet (IQR: 0.00, median: 0.00) and overbite (IQR: 0.50, median: 0.00) and arch dimensions (IQR: 0.30, median: 0.10). The largest measurement difference can be seen in the arch dimensions, while the smallest measurement difference is in the tooth widths

**Fig. 3:**
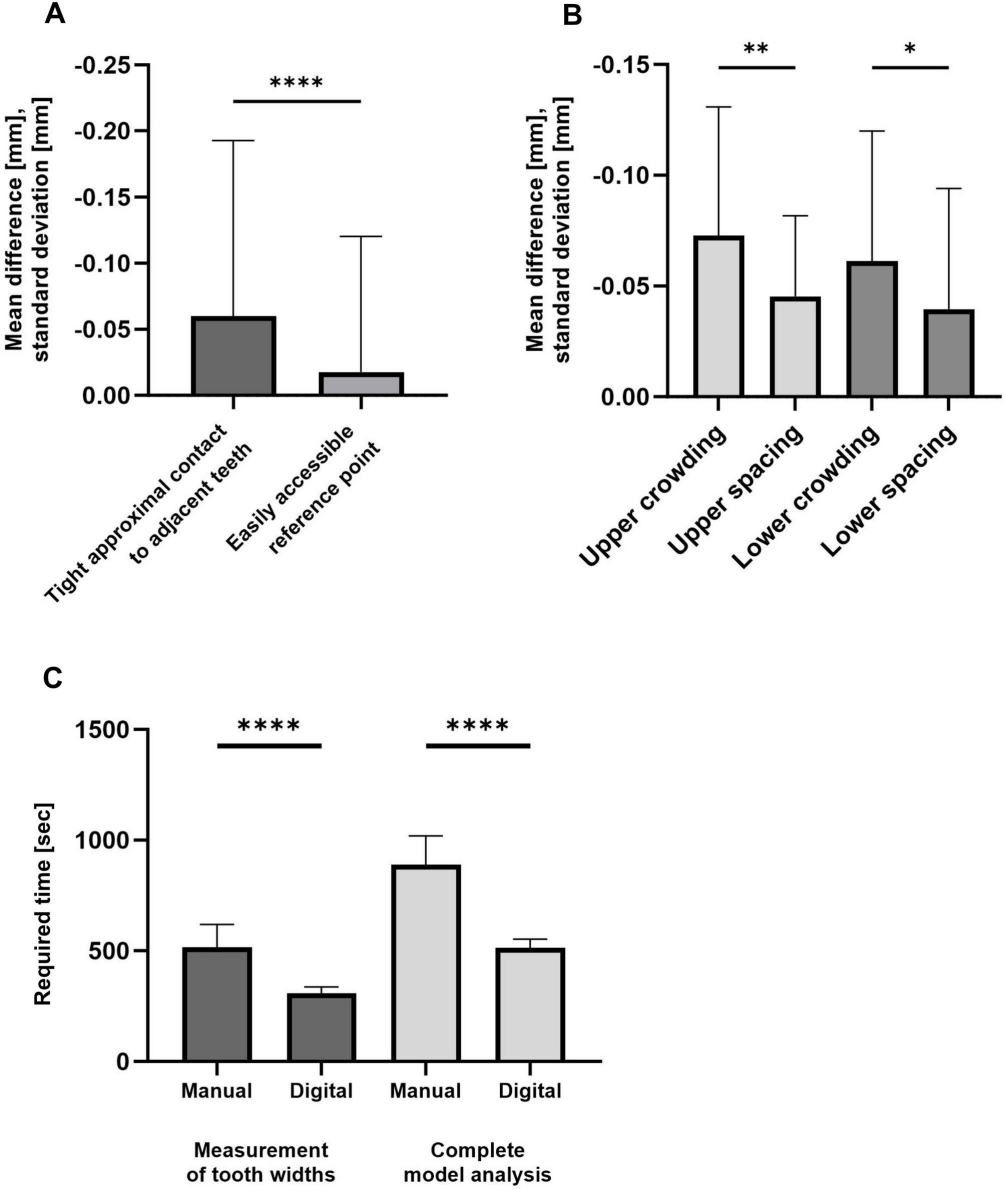
Differences between the digital and analogue measuring methods regarding the accessibility of the measuring points and the time required. **A**,** B**: Comparison of the deviation between digital and analogue tooth width measurement for teeth with easy access to the reference points (IQR: 0.10, median: 0.00) and close proximal contact to the adjacent teeth (IQR: 0.10, median: 0.00). The teeth with freely accessible measuring points show a smaller measurement difference between the digital and analogue measuring methods compared to the teeth with difficult-to-access reference points; upper crowding IQR: 0.143, median: -0.067; upper spacing IQR: 0.10, median: -0.042; lower crowding IQR: 0.125, median: -0.058; lower spacing IQR: -0.066, median: 0.042 **C**: Comparison of the time required [sec] between the digital and analogue measurement methods for the tooth width measurement (manual IQR: 157.50, median: 499.00; digital IQR: 39.70, median: 308.00) and the complete model analysis (manual IQR: 236.50, median: 889.00; digital IQR: 64.00, median: 513.55)

The plaster models were considered as the gold standard. The digital and analogue model analyses were compared directly based on all measurement parameters. In addition, the differences were calculated by subtracting the analogue value from the digital value. For further analysis the statistics software GraphPad PRISM Version 10.0.1/2023 (GraphPad Software Inc., San Diego, California, USA) was utilised. In order to examine the digital and analogue values for significant differences, all groups were first tested for normal distribution using the Shapiro-Wilk test. The direct comparison of the digital and analogue values (Table 2) was carried out using the Wilcoxon matched-pairs signed rank test and the bland-altmann plot (Supp.-Fig. 1a-k). The measurement differences between the digital and analogue values with regard to the different tooth groups (Fig. 2A), measurement criteria (Fig. 2B) and the amount of space in dental arch (Fig. 3B) were determined using the Kruskal-Wallis test. The accessibility of the measuring points (Fig. 3A) was statistically tested using the Mann-Whitney U-test. The comparison of the time required (Fig. 3C) was carried out using the paired T-test. *P* < 0.05 was considered statistically significant.

**Table 2:** Comparison of the results of the digital and analogue measurement methods. The tooth widths of the maxilla and mandible of 16–26, 36–46, the tooth crown height of 12–22, 32–42, the overjet and overjet, the midline shift in the maxilla and mandible and in relation to each other, the arch dimensions with anterior and posterior dental arch width for the maxilla and mandible, dental arch length and circumference were measured. The results show statistical significance using the Wilcoxon matched-pairs signed rank test, but are not clinically relevant due to their negligible size (see difference digital vs. analogue)

		digital							analog										
tooth width	*n* tooth pairs	Mean	SD	SEM	Lower 95% CI	Upper 95% CI	Median	IQR	Mean	SD	SEM	Lower 95% CI	Upper 95% CI	Median	IQR	*p*-value	Difference digital vs. analogue (*p*-value)	Median	IQR
16	242	10.47	0.6269	0.04030	10.40	10.55	10.50	0.70	10.43	0.6194	0.03982	10.35	10.51	10.40	0.80	< 0.0001	0.046 ± 0.134	0.00	0.10
15	212	6.809	0.4937	0.03390	6.743	6.876	6.80	0.70	6.735	0.4893	0.03361	6.669	6.802	6.70	0.70	< 0.0001	0.074 ± 0.139	0.00	0.10
14	227	7.116	0.4768	0.03164	7.054	7.179	7.10	0.60	7.045	0.4896	0.03250	6.981	7.109	7.00	0.60	< 0.0001	0.071 ± 0.130	0.00	0.10
13	223	7.831	0.4738	0.03173	7.769	7.894	7.80	0.70	7.784	0.4620	0.03093	7.723	7.845	7.80	0.60	< 0.0001	0.047 ± 0.160	-0.10	0.10
12	231	6.833	0.6784	0.04464	6.745	6.921	6.90	0.80	6.787	0.6726	0.04425	6.700	6.874	6.80	0.80	< 0.0001	0.046 ± 0.124	0.00	0.10
11	245	8.765	0.6123	0.03912	8.688	8.688	8.70	0.80	8.713	0.6083	0.03886	8.637	8.790	8.70	0.80	< 0.0001	0.052 ± 0.133	-0.10	0.10
21	245	8.760	0.6391	0.04083	8.680	8.840	8.80	0.90	8.696	0.6151	0.03939	8.619	8.774	8.70	0.85	< 0.0001	0.064 ± 0.201	-0.10	0.10
22	235	6.751	0.6694	0.04367	6.665	6.838	6.80	0.80	6.714	0.6524	0.04256	6.630	6.798	6.80	0.80	< 0.0001	0.037 ± 0.117	0.00	0.10
23	226	7.817	0.4836	0.03217	7.753	7.880	7.80	0.70	7.779	0.4887	0.03251	7.715	7.843	7.80	0.80	< 0.0001	0.038 ± 0.116	0.00	0.10
24	226	7.131	0.4724	0.03143	7.069	7.192	7.20	0.60	7.066	0.4798	0.03192	7.003	7.129	7.10	0.60	< 0.0001	0.064 ± 0.117	0.00	0.10
25	216	6.770	0.4869	0.03313	6.705	6.835	6.80	0.575	6.697	0.4818	0.03278	6.632	6.761	6.70	0.60	< 0.0001	0.073 ± 0.141	-0.10	0.10
26	241	10,44	0.5796	0.03733	10.37	10.52	10.40	0.70	10,39	0.5840	0.03762	10.32	10.46	10.30	0.70	< 0.0001	0.054 ± 0.122	0.00	0.10
46	238	10,99	0.7087	0.04594	10.90	11.08	11.00	0.90	10,91	0.7289	0.04725	10.82	11.01	10.90	0.80	< 0.0001	0.079 ± 0.169	-0.10	0.10
45	216	7.318	0.6411	0.04362	7.232	7.404	7.30	0.575	7.227	0.6420	0.04368	7.141	7.313	7.150	0.60	< 0.0001	0.091 ± 0.151	-0.10	0.20
44	224	7.203	0.4726	0.03158	7.141	7.265	7.20	0.60	7.146	0.4593	0.03069	7.086	7.206	7.10	0.575	< 0.0001	0.057 ± 0.122	0.00	0.10
43	227	6.793	0.4391	0.02915	6.736	6.851	6.80	0.60	6.764	0.4307	0.02859	6.708	6.820	6.80	0.60	< 0.0001	0.030 ± 0.117	0.00	0.10
42	246	5.995	0.4416	0.02815	5.940	6.051	6.00	0.60	5.951	0.4271	0.02723	5.898	6.005	6.00	0.60	< 0.0001	0.044 ± 0.125	0.00	0.10
41	246	5.376	0.3947	0.02516	5.327	5.426	5.40	0.60	5.387	0.3883	0.02476	5.338	5.436	5.40	0.50	0.3427	− 0.011 ± 0.137	0.00	0.20
31	246	5.378	0.4052	0.02583	5.327	5.429	5.30	0.40	5.385	0.3976	0.02535	5.335	5.435	5.40	0.60	0.4468	− 0.008 ± 0.129	0.00	0.20
32	247	6.013	0.4180	0.02660	5.961	6.066	6.00	0.60	5.971	0.4131	0.02629	5.919	6.023	6.00	0.60	< 0.0001	0.043 ± 0.109	-0.10	0.10
33	228	6.843	0.4542	0.03008	6.783	6.902	6.90	0.60	6.801	0.4474	0.02963	6.743	6.860	6.80	0.60	< 0.0001	0.041 ± 0.092	0.00	0.10
34	221	7.218	0.4484	0.03017	7.158	7.277	7.20	0.50	7.153	0.4288	0.02884	7.096	7.210	7.20	0.50	< 0.0001	0.065 ± 0.142	0.00	0.10
35	213	7.342	0.4821	0.03303	7.277	7.407	7.30	0.60	7.245	0.4914	0.03367	7.179	7.311	7.20	0.60	< 0.0001	0.097 ± 0.146	0.00	0.20
36	234	11.03	0.6888	0.04503	10.94	11.12	11.00	0.80	10,95	0.7009	0.04582	10.86	11.04	10.95	0.83	< 0.0001	0.073 ± 0.197	-0.10	0.10
**crown height**																			
12	198	7.602	1.070	0.07601	7.452	7.752	7.50	1.30	7.650	1.112	0.07900	7.494	7.806	7.60	1.20	0.0481	− 0.048 ± 0.262	-0.10	0.30
11	214	9.423	1.026	0.07016	9.285	9.561	9.40	1.40	9.467	1.101	0.07524	9.319	9.615	9.50	1.50	0.1255	− 0.044 ± 0.295	0.00	0.30
21	211	9.393	1.004	0.06909	9.257	9.529	9.30	1.30	9.493	1.105	0.07609	9.343	9.643	9.40	1.40	< 0.0001	− 0.100 ± 0.285	-0.10	0.20
22	202	7.622	1.099	0.07734	7.470	7.775	7.60	1.60	7.725	1.156	0.08130	7.565	7.886	7.70	1.50	< 0.0001	− 0.103 ± 0.317	-0.10	0.30
42	201	7.447	0.9653	0.06809	7.313	7.581	7.40	1.25	7.571	1.130	0.08011	7.413	7.729	7.50	1.30	< 0.0001	− 0.132 ± 0.385	-0.10	0.20
41	205	7.608	0.8975	0.06268	7.484	7.731	7.70	1.20	7.802	1.061	0.07410	7.656	7.948	7.80	1.50	< 0.0001	− 0.194 ± 0.528	-0.10	0.30
31	199	7.669	0.8705	0.06171	7.547	7.791	7.60	1.00	7.811	1.006	0.07130	7.670	7.951	7.80	1.10	< 0.0001	− 0.142 ± 0.410	-0.10	0.40
32	202	7.482	0.9648	0.06788	7.348	7.616	7.50	1.20	7.644	1.105	0.07775	7.490	7.797	7.60	1.225	< 0.0001	− 0.162 ± 0.390	-0.10	0.30
**Overjet**	233	4.139	3.492	0.2288	3.689	4.590	4.10	3.50	4.027	3.431	0.2247	3.584	4.470	4.00	3.20	< 0.0001	0.106 ± 0.278	0.10	0.40
**Overbite**	232	3.036	3.003	0.1971	2.647	3.424	3.45	3.40	2.894	3.012	0.1978	2.504	3.283	3.35	3.50	< 0.0001	0.142 ± 0.399	0.10	0.30
**MLD UJ**	230	0.2313	0.7127	0.04699	0.1387	0.3239	0.15	0.60	0.1778	0.7605	0.05015	0.07902	0.2766	0.00	0.525	0.0045	0.053 ± 0.300	0.00	0.30
**MLD LJ**	227	0.05419	1.451	0.09632	-0.1356	0.2440	0.00	1.70	0.1193	1.496	0.09905	-0.07587	0.3145	0.10	1.70	0.0135	− 0.066 ± 0.462	-0.10	0.50
**MLD UJ-LJ**	227	-0.1872	1.479	0.09817	-0.3807	0.006213	-0.20	1.70	-0.06828	1.494	0.09916	-0.2637	0.1271	0.00	2.00	< 0.0001	− 0.119 ± 0.423	-0.10	0.40
**dental arch** ** width**																			
anterior UJ	165	33.62	3.492	0.2718	33.09	34.16	33.80	3.75	33.81	3.467	0.2699	33.28	34.35	33.90	3.80	< 0.0001	− 0.190 ± 0.232	-0.20	0.20
anterior LJ	194	26.10	2.355	0.1690	25.77	26.44	25.95	2.82	26.28	2.329	0.1672	25.95	26.61	26.10	2.50	< 0.0001	− 0.173 ± 0.233	-0.20	0.30
posterior UJ	242	50.65	3.528	0.2268	50.21	51.10	50.60	4.72	50.82	3.530	0.2269	50.37	51.27	50.80	4.72	< 0.0001	− 0.165 ± 0.232	-0.20	0.30
posterior LJ	228	51.26	2.917	0.1932	50.88	51.64	51.05	3.97	51.41	2.918	0.1932	51.03	51.79	51.35	4.05	< 0.0001	− 0.146 ± 0.283	-0.20	0.40
**dental arch ** **length**																			
UJ	237	36.38	3.108	0.2019	35.98	36.77	36.60	3.80	36.61	3.102	0.2015	36.21	37.01	36.80	3.60	< 0.0001	− 0.233 ± 0.269	-0.30	0.30
LJ	228	32.88	2.805	0.1858	32.52	33.25	33.00	3.30	33.09	2.794	0.1850	32.72	33.45	33.30	3.58	< 0.0001	− 0.204 ± 0.298	-0.30	0.40
**dental arch** ** girth**																			
UJ	240	74.36	5.750	0.3712	73.62	75.09	74.70	7.72	74.60	5.750	0.3712	73.87	75.33	74.80	7.80	< 0.0001	− 0.247 ± 0.340	-0.30	0.50
LJ	240	65.79	5.823	0.3759	65.05	66.53	66.00	6.38	66.03	5.794	0.3740	65.29	66.77	66.30	6.67	< 0.0001	− 0.244 ± 0.326	-0.30	0.40
**Bolton ratio**																			
**AR**	211	77.87	2.782	0.1915	77.50	78.25	77.82	3.58	78.09	2.730	0.1879	77.72	78.46	78.02	3.60	0.0010	0.219 (± 0.939)	0.16	1.05
**OR**	167	91.64	2.256	0.1745	91.30	91.98	91.56	3.04	91.64	2.235	0.1729	91.30	91.98	91.42	3.07	0.4589	− 0.003 (± 0.831)	-0,02	0.84

L 95% CI: Lower 95% CI of mean; IQR: interquartile range, p-value Wilcoxon matched-pairs signed rank test; UJ: upper jaw; LJ: lower jaw, MLD: midline deviation, AR: anterior ratio of Bolton, OR: overall ratio of Bolton

## Results

247 pseudonymised patient cases from the study center Clinic for Orthodontics of the University Hospital RWTH Aachen, 129 female and 118 male patients with an average age of 16.67 +- 9.49 years with mixed or permanent dentition, were included in this retrospective cohort study and examined using a digital, semi-automated orthodontic model analysis with the OnyxCeph software. The digital measurements were compared with the gold standard of analog model analysis.

The different methods agree to within 0.3 mm for direct measurements and to within 0.66 for computed space parameters in model analysis. The results of the digital measurement method could be recorded in a shorter time.

### Power

Power analyses with α (one-tailed) = 0.05, effect size = 0.23 and power (1 - β) = 0.95 resulted in a minimum sample size of *N* = 206. Therefore, we included a total of 247 pseudonymised cases from patients being treated at the University Hospital Aachen, RWTH.

### Reliability

For digital measurements, an intraclass correlation coefficient (ICC) between 0.996 and 0.9995 was calculated for the different measurements. For repeated measurements performed on plaster models, the results varied between 0.991 and 0.9997. The agreement of the digital measurements by the second examiner was rated from 0.946 to 0.997.

### Accuracy of measuring tooth size

**Fig. 4:**
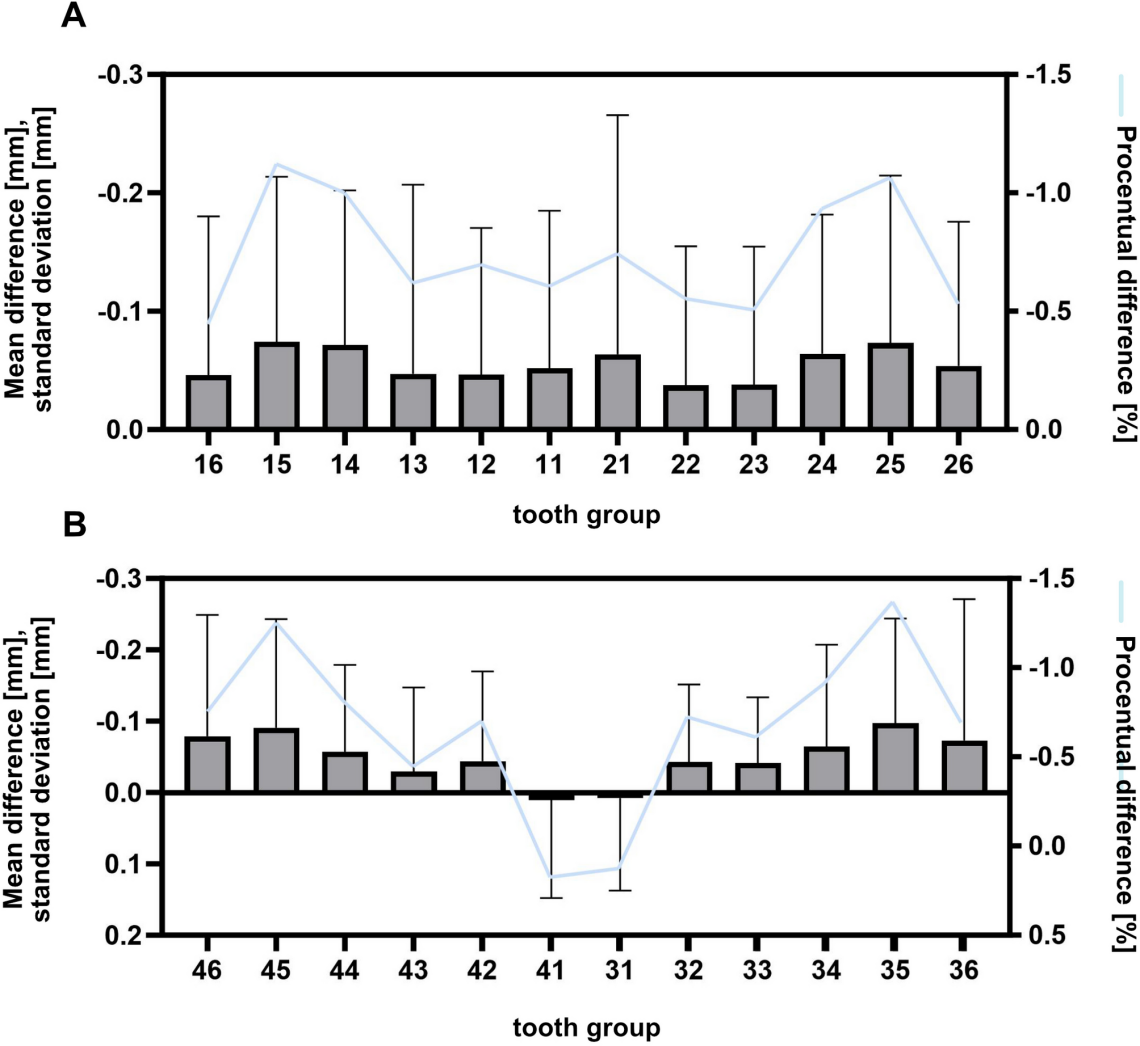
Differences between the digital and analogue measuring methods regarding the tooth groups, the accessibility of the measuring points and the time required. **A**: The average absolute (left y-axis) and percentage (right y-axis) measurement difference between the digital and analogue measurement methods in relation to the upper jaw with teeth 16–27 and **B**: in relation to the lower jaw with teeth 36–46, with the premolars showing the greatest deviation in comparison

A total of 5497 digitally (partly automatically) and analogously measured tooth widths from 247 cases were compared. Table 2 provides an overview of the digital and analogue measurement results for the tooth widths in the upper (teeth 16–26) and lower jaw (teeth 36–46), the Bolton ratio AR and OR, the crown height of teeth 12–22, 32–42, the overjet and overbite, the midline shift in the upper and lower jaw and the deviation from each other, the arch dimensions with anterior and posterior arch width, the arch length and circumference in the upper and lower jaw. There was an absolute average difference of 0.053 mm (+-0.025 mm) between the digital and analogue tooth width measurements. Thus, the digital method seems to provide larger values of about 0.023 mm on average. A Wilcoxon matched-pairs signed rank test was conducted, which revealed statistically significant differences between the values obtained using the two methods. The mean absolute and percentage differences for teeth 16–26 in the maxilla and 36–46 in the mandible are shown in Fig. 4A, B. Furthermore, the differences that occurred were analysed and specified further by tooth type regarding molars, premolars, canines and incisors (Fig. 2A) and based on the different measurement criteria (facewidth, crown height, overjet/overbite, arch dimensions) (Fig. 2B). It was found that premolars show a higher deviation between the measurement on the digital model and the plaster model than molars, canines or incisors (Fig. 2A). The largest measurement difference can be seen in the arch dimensions, while the smallest measurement difference is in the tooth widths (Fig. 2B).

### Bolton ratio

Based on our inclusion criteria for model selection and mixed dentition models or tooth agenesis, Bolton’s Analysis was performed using each method, and the values were examined to see if the values differed (Table 2). AR could be determined for 211 patients and OR for 167 of the 247 patients. The mean absolute difference was relative 0.57 for AR and 0.54 for OR. When the calculated Bolton ratios of all patients were compared between the digital and analogue methods, significant differences were found for AR. However, there was no significant difference for OR.

### Comparison of measuring techniques

Bland-Altman plots were constructed for the values obtained by both methods (Supp.-Fig. 1a-k). These plots contain ‘limits of agreement’ that define a range that covers 95% of the differences between the values obtained by different methods or repeated measurements. The mean differences and limits of agreement resulting from the comparison of the two measurement methods (caliper and software) are shown in Supp.-Fig. 1a-k. The mean difference between all individual tooth measurements (tooth width, crown height) is + 0.05 mm for tooth width with 95% limits of agreement of + 0.31 and − 0.22 mm and − 0.12 mm for crown height with 95% limits of agreement of + 0.61 and − 0.84 mm. The measurements of inter- and intramaxillary individual distances (overjet, overbite, midline deviation, arch width, arch length, arch girth) showed the smallest mean difference of -0.03 mm with 95% limits of agreement of + 0.77 mm and − 0.83 mm for the midline deviation and the largest mean difference of -0.25 mm with 95% limits of agreement of + 0.41 mm and − 0.90 mm for the arch girth. The cumulative measurements showed a mean difference of -0.22 mm for the anterior Bolton ratio with greater 95% limits of agreement than the single tooth measurements and individual distances of + 1.63 mm and − 2.06 mm. The total Bolton ratio showed a mean difference of -0.25 mm with 95% limits of agreement of + 1.64 mm and − 1.63 mm.

### Risk factors increasing the deviation of measurements

When comparing digital and analogue measurements, risk factors that can lead to increased measurement differences must be considered. As shown in Fig. 4A, crowding in an arch appeared to significantly increase the difference between digital and analogue measurements of tooth width in the maxilla and mandible. Crowding was defined as having a negative result when performing the spacing analysis. All other cases with a positive result were included in the spacing group. The group with present crowding in the maxilla or mandible shows an increased mean difference of 0.076 mm, compared to 0.044 mm in the maxilla and 0.057 mm in the mandible with spacing. In addition, an individual evaluation of each tooth was also documented. The observed differences between the two measurement methods were compared between two groups according to access to the reference point and the results are shown in Fig. 4B. Teeth with tight approximal contact showed a significantly higher deviation between the two measurement methods, averaging approximately 0.059 mm, than teeth with freely accessible reference points, averaging approximately 0.027 mm.

### Time saving

A comparison was conducted between the required time for the digital and analogue methods, separately for tooth width measurement and complete model analysis. The results are provided in Fig. 4C. The mean time required for the measurement of tooth widths 16–26 and 36–46 on plaster models was 8:36 min. A significant reduction in the measurement time was observed in comparison with digital determination, with a reduction to 5:09 min. Consequently, a time saving of 3:27 min was achieved. The required time for the complete analysis of the plaster models was also analysed. This included the measurement of tooth widths and heights (12–22 and 32–42), as well as anterior and posterior arch widths, arch lengths, perimeter, overjet and overbite were considered. The digital model analysis was completed in a mean time of 8:33 min, whereas the manual analysis of plaster models required a mean time of 14:48 min. Consequently, a reduction in the required time of 6:15 min was demonstrated. The observed differences in required time were statistically significant.

## Discussion

In this study, we investigated the accuracy, reliability and efficiency of digital model analysis based on virtual models digitised with the model scanner *orthoXscan* (Dentaurum GmbH & Co. KG, Ispringen, Germany) and analysed with the software *OnyxCeph*^*3TM*^ (Image Instruments GmbH, Chemnitz, Germany), in comparison to conventional manual model analysis on plaster models and a digital calliper. Our findings indicate that partly automated digital cast analysis is a timesaving and therefore highly efficient process and represents an accurate and reliable alternative to traditional manual cast analysis, which are of particular interest to orthodontists.

A comparison of the measurement of tooth width between digital and plaster models revealed that the partially automated digital method delivered by an amount of 0.023 mm resulted in increased values according to Kardach et al. [17]. Studies have shown varying results in measuring tooth width, with some finding no statistical difference but a slight decrease in digital values up to 0.023mm [18]. Regardless of the statistical significance observed in the discrepancy between analogue and digital methods in measuring tooth width, the results obtained with the Bolton analysis demonstrated no significant differences in terms of OR [18]. In contrast to our results, Lo Giudice et al. found no significant differences in OR [19]. Conversely, the differences in AR were not significant, which is not consistent with our results and those of Kim et al. [20]. The lack of discrepancies in the overall Bolton analysis can be attributed to the assumption of a consistent bias in tooth width. After accounting for this assumption, the proportional value of OR effectively compensates for the differences in single tooth width [11, 17, 18]. In some cases, especially in the measurement of crown height, the digital measurement process proved to be challenging due to the occurrence of boundary blurring during the reconstruction of attached gingiva in the segmentation process. This resulted in difficulties in accurately defining the cervical reference point. Consequently, this can result in a higher absolute mean difference of 0.241 mm regarding to the crown height compared to Keating et al.´s (0.1 mm) and Camardella et al.´s (0.17 mm) findings [21, 22]. In this case, other measuring tool as the “calliper measurement’’ could be more accurate to measure the crown height of these teeth. Furthermore, is also important to note that the measurement ranges for tooth width and height are similar, but that for crown height, there is a nearly four times higher deviation. Although the discrepancy is below the 0.3 mm threshold [5], it is not clinically relevant. In our study the arch dimensions showed measurements with a significantly decreased difference of 0.297 mm. In disagreement to the literature, increased values for arch widths and arch perimeter were found [23]. This result can be attributed to potential misinterpretations during the selection of reference points (centre of the cusp), particularly in cases where teeth exhibit attrition on the reference cusp. The estimation of the arch perimeter in the context of space analysis represents a key aspect of the diagnostic and planning processes employed in orthodontics. Arch perimeter estimation, such as intermolar and intercanine widths, displayed significant differences between plaster and digitised models. The clinical relevance of the detected results was questioned by the authors [23]. This type of measurement revealed in our study the greatest absolute difference between analogue and digital measurements. It is important to note that the results should be interpreted in the context of the large distances that were analysed. Therefore the percentage difference is the smallest of all the measurements that were conducted, and it is evident that the results are not clinically significant when compared to the literature [23]. In contrast to other studies, the measurement of overjet was found to be statistically different in our study [11, 18]. Similarly, the measurement of the overbite differed with statistical significance in other studies, amounting to 0.3 mm or 0.49 mm, respectively [11, 18]. In contrast, our determined mean difference was much smaller, with 0.236 mm for the overbite. In the aforementioned studies [11, 18], the discrepancy was deemed to be of no clinical significance despite statistical significance. This may be indicative of the similarly insignificant discrepancy observed in our study.

In conclusion, this prompts a fundamental debate about the clinical significance of statistical significance. A perusal of Table 2 reveals that all the observed differences in measurements are statistically significant. However, it is not immediately evident whether these differences are automatically clinically relevant for orthodontists. To address this fundamental question, a review of the relevant literature was conducted. It was recommended by Bishara et al. that a maximum difference of 0.2 mm should be allowed for repeated measurements carried out by a single examiner. The discrepancy in measurements for tooth width and overjet between the digital and plaster methods in our study was below the threshold levels required for repeated measurements with the same method, as previously stipulated [24, 25]. Another author has stated that for a digital model in orthodontics, 0.3 mm is set as the threshold of required accuracy [5]. Deviation for non-calculated measurements of up to 0.5 mm is considered to be clinically irrelevant in the literature [11, 26]. All the differences in the measurements presented in Table 2 are below this value, thus satisfying the requirements. When analysing the measurement results, it can be assumed that the statistically significant but at the same time clinically irrelevant measured values should not significantly influence the treatment decision. It may be necessary to make an exception for the crown height. As previously stated, there are discrepancies between the findings of different studies, which raises the question of what factors are responsible for the observed discrepancies in study results.

When analysing the Bland-Altman plot, it should be noted that both limits of agreement increase when measuring individual distances (with the exception of arch width), and even more so when cumulative measurements are made. Whether this variability is clinically acceptable cannot be easily answered. The tolerance limit of ± 0.5 mm postulated in the literature [11] suggests that the non calculated measurements of tooth and arch widths are clinically acceptable. At the same time, it should be noted that more complex measurements (arch length or girth) and cumulative measurements were not included in the above study and therefore the tolerance limit of ± 0.5 mm may not be applicable. Our results suggest a complexity-dependent proportionality. The more complex and extensive the measured structure (from individual teeth to dental arches to commutative groups of teeth), the greater the percentage deviations. This suggests a proportional relationship in which measurement uncertainty increases with the size and complexity of the measured structure. In general, it can be said that the mean of difference/distortion is low and the relatively small range of the limits of agreement in relation to the complexity of the measurements indicates a low variability of the differences.

As described above, there are inconsistencies between different investigations, which leads to the question what factors are causing differences between study results.

One of the factors that can affect the accuracy of a measurement is the level of experience of the observer. In the context of our study, the rater was experienced, familiar with the software being used, and completed all measurements in the allotted time of two months. The research group has many years of experience in digital measurement with the OnyxCeph software, due to regular internal training by the developers of the software program itself, previous digital studies and prior research results, the daily use of digital analysis of patient cases in clinical orthodontic practice and to support, continuously optimise and further develop the software tool. It can be observed that observers with more experience tend to produce more consistent readings when undertaking repeated measurements, although this may not always be the case [27]. However, the results of repeated measurements can also vary, even if performed by the same observer. This is particularly true when the interval between repeated measurements is extended [28]. Furthermore, it is important to acknowledge that the process of marking reference points in a three-dimensional model on a two-dimensional computer screen may present certain challenges [29]. This is due to the fact that points may be evaluated differently depending on the adjusted point of view, which can be particularly problematic when working with complex models. The challenge lies in identifying an identical reference point in both analogue and digital forms [6, 30].

In the present study, teeth exhibiting close approximate contact with neighbour teeth and the presence of crowding demonstrated an elevated discrepancy. Regarding the issue of point positioning, callipers are unable to attain the maximum mesiodistal diameter of teeth, especially in the presence of crowding. Moreover, impression materials are unable to accurately reproduce the space between crowded teeth in plaster casts [31]. In contrast, software solutions for digital model analysis offer a range of functions, including zooming and view rotation particularly at proximal contacts, even in the presence of crowding. Conversely, one source for bias in this context can be the poor resolution of the approximate area between teeth in the plaster model, in which the software is making a proposal for the missing information delineating the merging teeth surfaces of adjacent teeth. This approach may diverge from that of the examiner when the calliper is applied. A review of the literature reveals that there are discrepancies between analog and digital measurements, which are attributed to varying degrees of crowding [19, 32, 33]. The authors also propose that these discrepancies have clinical implications [33]. Furthermore, it would be beneficial to discuss whether the selection of traditional plaster models can be considered the gold standard and using an intraoral scanner by a trained user may avoid this bias.

In the analysis of the time required to perform the measurements, a significant reduction was observed using the digital method, which is consistent with other studies [8–10]. Looking at the significant results of timekeeping that have been studied for more than 10 years, the technology has improved considerably. Today, the software places the reference points automatically and accurately, and only needs to be checked and adjusted by the user, which can explain another time saving in our study. The first step in the process chain, which includes the traditional impression or direct digital intraoral scanning, was not studied here. Recent studies on chairside time shows a differentiated picture of the time efficiency of digital workflows, which is determined by complex interactions between technology maturity, user experience, and clinical application context, which reveal situation-dependent delay potentials [34–36]. Again, the literature is clear on the benefits and efficiency of digital intraoral scanning, which should be kept in mind [37–39].

The comparison of measurement methods on plaster models represents a limitation in terms of bias, as the use of intraoral scans only would allow a more accurate analysis. For users with non-specialised digital equipment, the results may be of limited value.

## Conclusion

In conclusion, the present study of orthodontic model analysis using plaster casts and a calliper and virtual models obtained with orthoX^®^scan and analysed with OnyxCeph3TM shows that partially automated digital cast analysis is an accurate, highly efficient and time-saving alternative to traditional manual cast analysis. The methods agree to within 0.3 mm for direct measurements and to within 0.66 for calculated space parameters in model analysis. Caution should be exercised with some calculated values due to bias associated with the assumption of cumulative error. With the exception of crown height, the measurement differences are not clinically relevant. Improvements in measurement accuracy and automatic reference point detection are expected as the technology develops. It is therefore necessary and appropriate for clinicians to gain further experience with digital systems. The further transition to automated and digital methods will be of increasing interest in future clinical orthodontics.

## Electronic supplementary material

Below is the link to the electronic supplementary material.

Supplementary Material 1

## Data Availability

The data underlying this article will be shared on reasonable request to the corresponding author.
