# Supplementary material for: Validation of a digital, partly automated three-dimensional cast analysis for evaluation of orthodontic treatment assessment
Source: Head Face Med. 2025 May 8;21:36. doi: 10.1186/s13005-025-00515-8 (PMC12060358; doi:10.1186/s13005-025-00515-8)
Supplement: Supplementary file 1 — Supplementary Material 1 [file 13005_2025_515_MOESM1_ESM.docx]

**Validation of a digital, partly automated three-dimensional cast analysis for evaluation of orthodontic treatment assessment**

Franziska A. Coenen^1*^, Norbert A. Lang^1*^, Julia Vorloeper^1^, Christian Niederau^1^, Rogerio B. Craveiro^1^ Isabel Knaup^1*^, Michael Wolf^1*^

^1^ Department of Orthodontics, RWTH Aachen University, Aachen, Germany

^*^  These authors contributed equally.

**Supplementary data**

**a b**

**
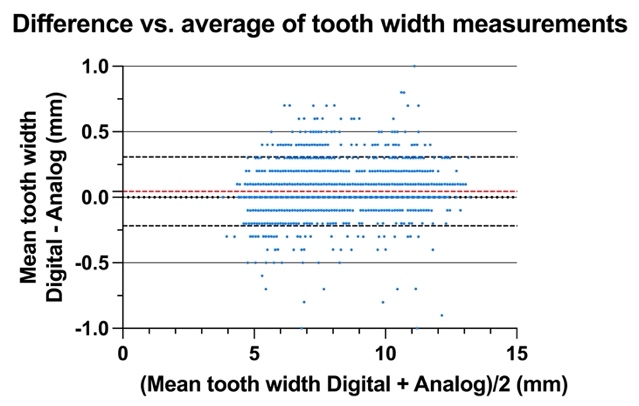

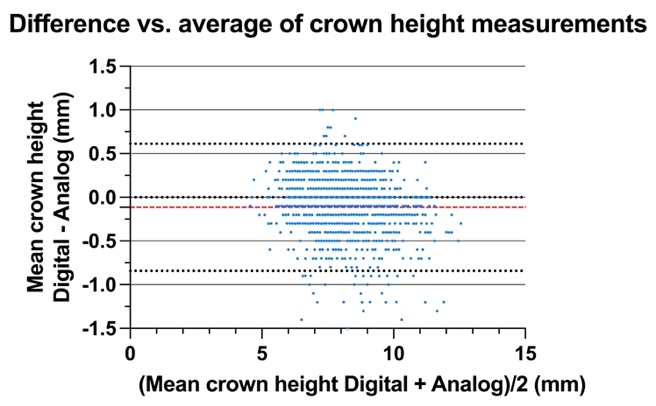
**

**c d**

**
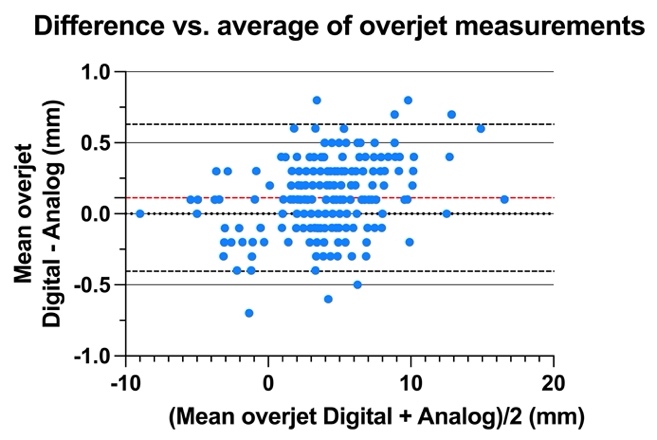

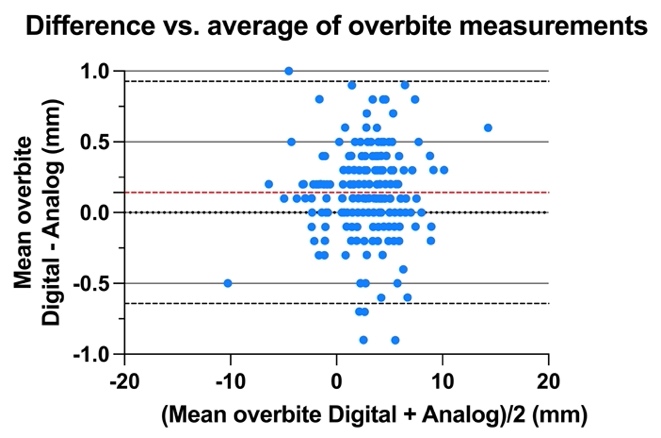
**

**e f**

**
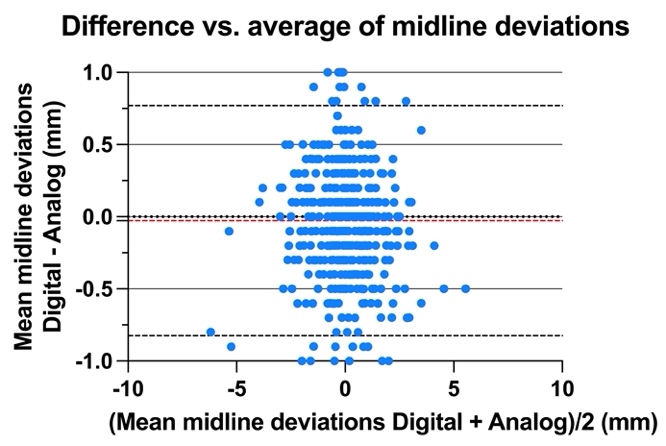

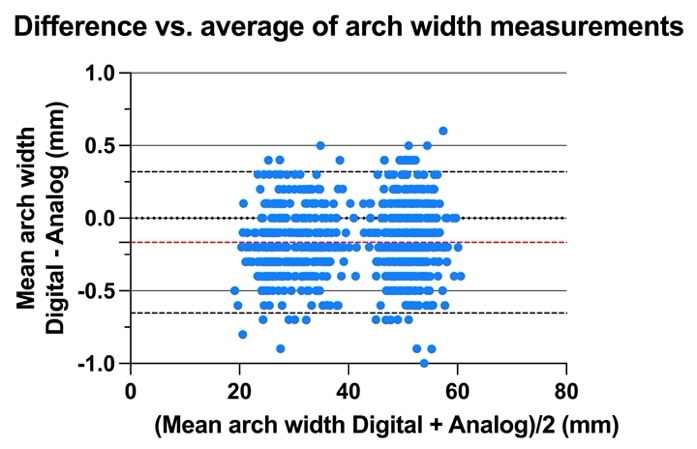
**

**g h**

**
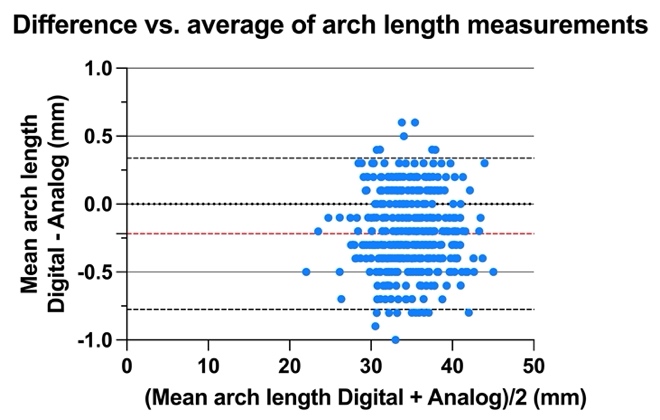

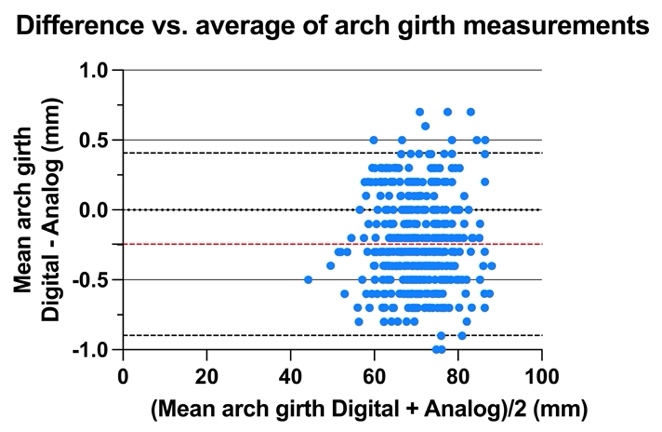
**

**i k**

**
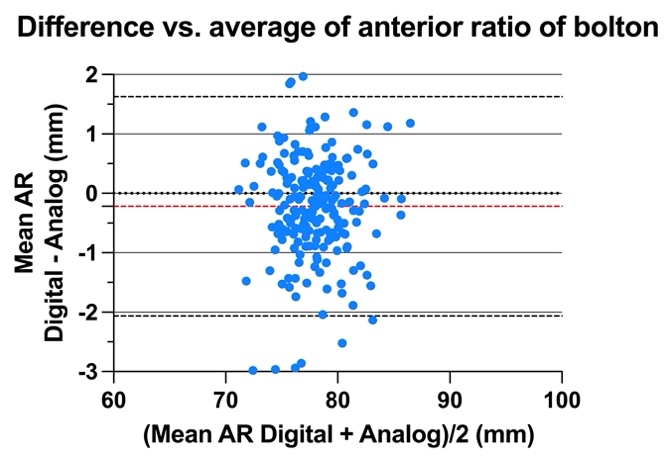

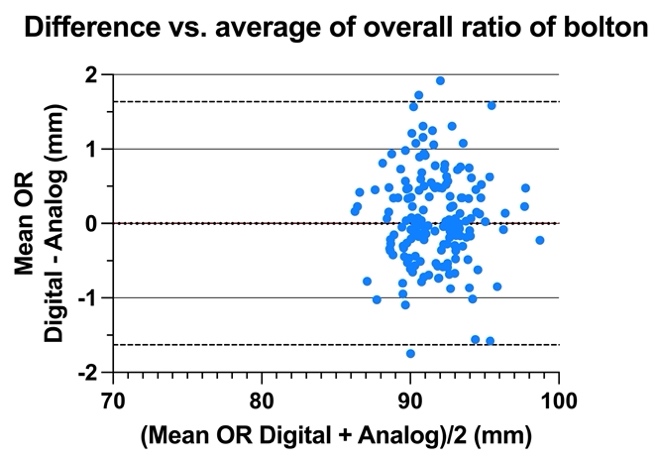
**

**Supp.- Fig. 1 a-k**

Comparison of the results of the digital and analogue measurement methods (blue points) using the Bland-Altmann plot. The tooth widths of the maxilla and mandible of 16-26, 36-46, the tooth crown height of 12-22, 32-42, the overjet and overbite, the midline shifts (in the maxilla and mandible and in relation to each other), the arch dimensions with anterior and posterior dental arch width for the maxilla and mandible, dental arch length and circumference and calculated means (AR, OR) were measured. The mean of the difference/ bias (red dashed line); upper/lower 95% limits of agreement (black dashed lines).
